# Magnetocaloric and Giant Magnetoresistance Effects in La-Ba-Mn-Ti-O Epitaxial Thin Films: Influence of Phase Transition and Magnetic Anisotropy

**DOI:** 10.3390/ma15228003

**Published:** 2022-11-12

**Authors:** Marwène Oumezzine, Cristina Florentina Chirila, Iuliana Pasuk, Aurelian Catalin Galca, Aurel Leca, Bogdana Borca, Victor Kuncser

**Affiliations:** 1 Laboratoire de Physico-Chimie des Matériaux, Université de Monastir, Monastir 5019, Tunisia; 2 National Institute of Materials Physics, 077125 Magurele, Romania

**Keywords:** perovskite manganite, epitaxial thin films, magnetoresistance, magnetocaloric effect, anisotropy

## Abstract

Magnetic perovskite films have promising properties for use in energy-efficient spintronic devices and magnetic refrigeration. Here, an epitaxial ferromagnetic La_0.67_Ba_0.33_Mn_0.95_Ti_0.05_O_3_ (LBMTO-5) thin film was grown on SrTiO_3_(001) single crystal substrate by pulsed laser deposition. High-resolution X-ray diffraction proved the high crystallinity of the film with tetragonal symmetry. The magnetic, magnetocaloric and magnetoresistance properties at different directions of the applied magnetic field with respect to the *ab* plane of the film were investigated. An in-plane uni-axial magnetic anisotropy was evidenced. The LBMTO-5 epilayer exhibits a second-order ferromagnetic-paramagnetic phase transition around 234 K together with a metal–semiconductor transition close to this Curie temperature (T_C_). The magnetic entropy variation under 5 T induction of a magnetic field applied parallel to the film surface reaches a maximum of 17.27 mJ/cm^3^ K. The relative cooling power is 1400 mJ/cm^3^ K (53% of the reference value reported for bulk Gd) for the same applied magnetic field. Giant magnetoresistance of about 82% under 5 T is obtained at a temperature close to T_C_. Defined as the difference between specific resistivity obtained under 5 T with the current flowing along the magnetic easy axis and the magnetic field oriented transversally to the current, parallel and perpendicular to the sample plane, respectively, the in-plane magneto-resistance anisotropy in 5 T is about 9% near the T_C_.

## 1. Introduction

Significant attention has been given in recent years to the search for new-generation device materials for magnetic storage technology and spintronics. Strongly correlated materials consisting of thin manganite films with a perovskite structure have been in the research spotlight because of their combination of spin, charge, orbit and lattice degrees of freedom [1,2]. Nevertheless, epitaxial thin films of this type demonstrate great potential for multifunctional device applications, such as magnetic refrigeration [3,4,5,6,7], spintronics and faster reading devices [8,9]. Such applications can be improved by the astonishing electronic specificities concomitant with giant magnetoresistance (GMR) and a large magnetocaloric effect (MCE). Moreover, the magnetic anisotropy and anisotropic magnetoresistance (AMR) properties of these materials have also attracted attention [10,11]. MCE research today is limited to bulk and single crystal materials, but studies on epitaxial rare-earth oxide thin films for MCE are very challenging. Thus, new fundamental studies will contribute to a deep understanding of the MCE of thin films. The AMR effect on perovskite manganites has been studied by a few research groups, indicating a peak in temperature dependence of the AMR near the Curie temperature T_C_ [12]. From the scientific point of view, fabrication of thin films of high structural quality and convenient and reliable control of their magneto-transport properties is crucial for making performant magneto-resistive devices. Note that many studies on mixed valence perovskite manganites have been focused on the partial substitution of manganese (electron-doped) with various metallic elements owing to significant changes in their magnetic and magneto-transport properties. Among the most cited microscopic mechanisms underlying the fascinating observations of macroscopic physical properties is the double-exchange (DE) interaction between Mn^3+^/Mn^4+^ and the Jahn–Teller-distorted ions Mn^3+^ [13,14]. Hence, the ferromagnetic ground state with metallic conduction arises from the hopping of the itinerant *e_g_* electron between neighboring Mn^3+^ and Mn^4+^ ions. Among the perovskite manganites, La_0.67_Ba_0.33_MnO_3_ (LBMO) has attracted particular attention due to its high Curie temperature of 345 K. Unlike bulk materials, few reports on the substitution of manganese with various metallic elements in manganite epilayers are available [15,16,17]. The substitution with the non-magnetic Ti^4+^ (d^0^) of the magnetic ion Mn^4+^ will causes a sudden break of the ferromagnetic Mn^3+^-O-Mn^4+^ interactions without any ferromagnetic compensation, allowing the fine tuning of T_C_ toward lower temperatures [18]. Using pulsed laser deposition (PLD), high-quality epitaxial thin films of La-Ba-Mn-Ti-O can be obtained, with pronounced properties such as MCE [19] and GMR [15]. Due to a strong shape anisotropy of thin films, it can be anticipated that the easy axis of magnetization will lie in the plane of the films, if the strain and surface/interface anisotropies are negligible. In this manuscript, the La_0.67_Ba_0.33_MnO_3_ system with substituent Ti cations at the Mn-site was chosen (5% of Mn is replaced by Ti). The film was successfully epitaxially grown on a SrTiO_3_(001) single crystalline substrate by PLD.

Here, we report a study of the temperature dependence of magnetization (M), magnetic entropy change (ΔS_M_), resistivity (ρ) and magnetoresistance (MR) under different directions of the applied magnetic field versus the *ab* plane of the epitaxial film. An increased response of such magneto-functionalities was evidenced for the in-plane orientations of the applied magnetic field.

## 2. Materials and Methods

The epitaxial La_0.67_Ba_0.33_Mn_0.95_Ti_0.05_O_3_ (referred to as LBMTO-5) thin film is grown by pulsed-laser deposition (PLD) on SrTiO_3_ (STO) with a (001) orientation. The detailed LBMTO-5 target material and LBMTO-5 film fabrication method is described in detail in a previous work [19]. HRXRD (High Resolution X-Ray Diffraction) patterns are measured with Cu K_α1_ radiation (1.5406 Å wavelength) using a D8 Discover diffractometer from Bruker AXS (Billerica, Massachusetts (MA), United States) in the 2θ-ω, φ-scan, and reciprocal-space mapping modes (RSMs). These structural characterizations permit us to determine the pseudocubic out-of-plane and in-plane lattice parameters of the films and to confirm the quality of the epitaxy. The magnetic properties were investigated by using a SQUID-Superconducting Quantum Interference Device magnetometer (MPMS 7T, Quantum Design, San Diego, California (CA), USA). According to our previous experience on the optimization of the magnetocaloric effects, the isothermal magnetizations were measured with the applied magnetic field along the in-plane directions. Temperature- and angle-dependent resistivity were investigated under various magnetic fields by using a Physical Property Measurements System (PPMS 14 T, Quantum Design, San Diego, California (CA), USA). In these measurements, the current was always flowing along the easy axis of magnetization specific to the rectangular-shaped sample, whereas the magnetic field was applied perpendicular to the sample plane or parallel to it, but always perpendicular to the current direction.

## 3. Results and Discussion 

### 3.1. LBMTO-5 Films Structure

**Figure 1:**
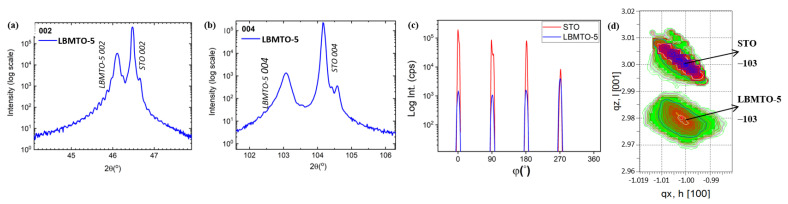
Diffractograms of LBMTO-5 thin film deposited on a STO(001) substrate. (**a**,**b**) Typical 2θ-ω scans showing the 002 and 004 peaks; (**c**) Azimuth φ-scan on the {103} skew planes of STO substrate and LBMTO-5; (**d**) asymmetric RSMs around the −103 node.

Figure 1a,b presents typical 2θ-ω diffractograms of LBMTO-5 thin films deposited on the STO(001). The pattern shows a two sets of LBMTO-5 diffraction peaks 002 and 004 together with those from the substrate, indicating that the films have a strong out-of-plane texture. The visible Pendellosung fringes indicate a smooth film/substrate interface and high crystal quality. The results of coherence length along [002] revealed a well-defined film thickness of 97 nm. The crystalline structure of the target material is rhombohedral, while the film is tetragonal due to substrate influence. The calculated *c*-axis parameter of the LBMTO-5 thin film is 3.935 Å. Figure 1c shows the corresponding RSMs around the asymmetric (−103) node revealing that the in-plane constant lattice of LBMTO-5 thin film is identical to that of the STO substrate. The epitaxial growth of the LBMTO-5 manganite on the STO substrate was confirmed by performing the XRD azimuth scans (ϕ-scans) on the {103} planes family of both substrate and thin films (Figure 1d). Each of the LBMTO-5 and STO exhibit four peaks, separated by 90°, suggesting ‘cube-on-cube’ epitaxial growth (presented in Supplementary Materials, Figure S1).

### 3.2. Magnetic and Magnetocaloric Effect Studies

**Figure 2:**
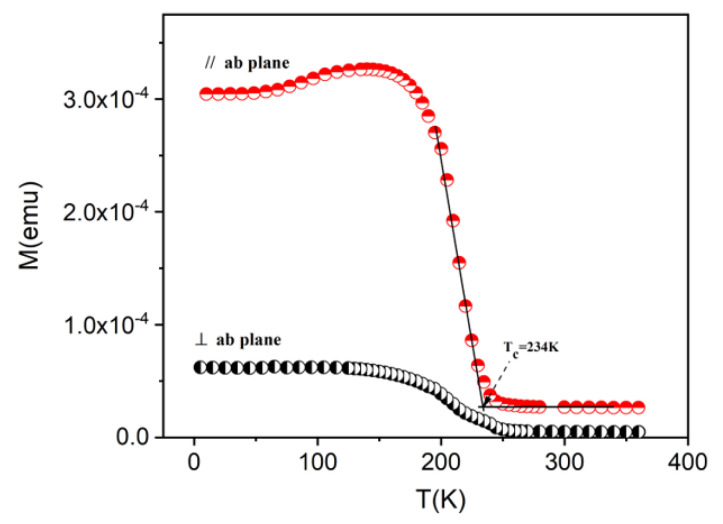
Plots of the field-cooled magnetization versus temperature of a LBMTO-5 thin film, measured under a magnetic field of 0.01 T parallel and perpendicular to the *ab* plane.

The magnetic characterization was realized on the 97 nm LBMTO-5/STO epitaxial thin film grown on the STO(001) substrate. Firstly, we measured the temperature dependences of field-cooled magnetization (M-T curves) in a magnetic field H with intensity of 100 Oe (equivalent of 0.01 T induction) applied along in-plane (H//film) and out-of-plane (H⊥film) directions, respectively. As shown in Figure 2, the in-plane magnetization values are higher than the ones measured in the out-of-plane configuration, with a maximum of about 5 times at 5 K. This observation supports the anisotropy with a preferential in-plane magnetic easy axis of the epitaxial thin film. The ferromagnetic–paramagnetic (FM–PM) transition of the LBMTO-5 film grown on the STO substrate can also be observed from these curves. The value of the Curie temperature T_C_ is found to be around 234 K. This was estimated with much higher precision in the in-plane geometry at the intersection point of the two tangents to the iso field curve that bounds the transition temperature, compared with the perpendicular geomerty (see Figure 2). This experimental value is slightly lower than the 264 K value found in the unstressed powder [18] and the 295 K value found in LBMTO-2/STO epitaxial thin films [15]. Similar to the bulk case, it can be seen that an extremely low doping (5% atomic percentage) with the non-magnetic Ti^4+^ replacing Mn^4+^ causes a sudden break of the ferromagnetic Mn^3+^-O^2−^-Mn^4+^ interactions without any ferromagnetic compensation. Furthermore, it is important to emphasize the role of in-plane compressive strain for stretching MnO_6_ octahedra in the out-of-plane direction, which induces a T_C_ shift to lower temperatures [20].

**Figure 3:**
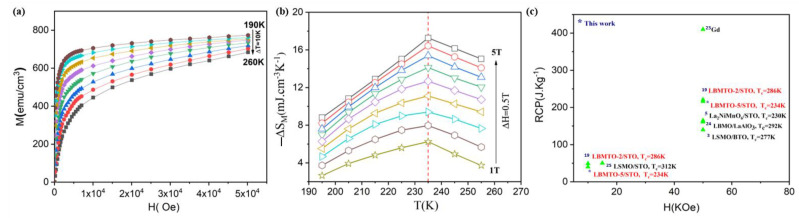
The magnetic properties and magnetocaloric effect of the LBMTO-5/STO(001) film. (**a**) Isothermal magnetization curves measured at different temperatures around T_C_. Inset shows the Arrott plot of μ_0_H/M versus M^2^. (**b**) Temperature dependence of the magnetic entropy change (ΔS_M_) under different applied magnetic fields. (**c**) Relative cooling power (RCP) as a function of the applied magnetic field compared with several other magnetic refrigerants thin films from the literature. The corresponding references [3,5,19,23,24,25] are marked in the left side of each material.

To evaluate the MCE characteristics of the present thin films, magnetic field dependent isothermal magnetizations were measured in-plane geometry, under fields up to 5 T induction (5 × 10^4^ Oe intensity) in the temperature range from 190 to 260 K with a temperature step of 10 K (see Figure 3a). All the M-H data have been corrected by subtracting the diamagnetic background (dominated by the substrate STO). The shape of the magnetization curves near T_C_ is typical of a second-order transition as usually observed in titanium-substituted (La,Ba)MnO_3_ manganites [19].

Based on the Banerjee criteria [21], the positive and negative sign of the slope of Arrott plots near T_C_ correspond to second-order magnetic phase transition (SOMPT) and first-order magnetic phase transition (FOMPT), respectively. Apparently, the positive slopes of Arrott plots in close proximity to T_C_ for LBMTO-5 /STO thin films, confirms SOMPT (see Supplementary Materials, Figure S2). On the other hand, maximum values of (−ΔS_M_)^max^ were predicted to show the proportional relationships ~ (μ_0_H/T_C_)^2/3^, which confirm the long-range ferromagnetic order (presented in Supplementary Materials, Figure S3). In a next step, the magnetic entropy change (ΔS_M_) was obtained using the Maxwell equation, which can be calculated for magnetization isotherms taken at discrete fields and temperatures [22]. The behavior of (−ΔS_M_) per unit volume is shown in Figure 3b.

The temperature dependence of the isothermal entropy change ΔS_M_ has a uniform distribution around T_C_ where the maximum is also reached. This maximum is increasing with the external magnetic field. One can see from Figure 3b that the peak position of (−ΔS_M_)^max^ remains unchanged, as an additional confirmation of SOMPT. Hence, our highest value of the (−ΔS_M_)^max^ obtained under a field induction change of 5 T, with incremental steps of 0.5 T, is 17.27 mJ/cm^3^ K (2.60 J/Kg K) and compares favorably with that obtained in LBMTO-2/STO epitaxial thin film. The maximum relative cooling power (RCP) is determined using the Wood and Potter method [26,27] and it is compared in Figure 3c with several other magnetic refrigerants’ thin films with T_C_ in the range of 230–321 K, as reported earlier in the literature. Similar to LBMTO-2/STO [19], our results indicate potential applications of the LBMTO-5 /STO film for micro-scale magnetic cooling.

Moreover, the maximum values of (−ΔS_M_)^max^ have proven to be proportional to (μ_0_H/T_C_)^2/3^ (see Figure S3 in Supplementary Materials), which confirms a long-range ferromagnetic order in the investigated epitaxial LBMTO-5 thin films below T_C._

### 3.3. Magnetotransport and Magnetoresistance

**Figure 4:**
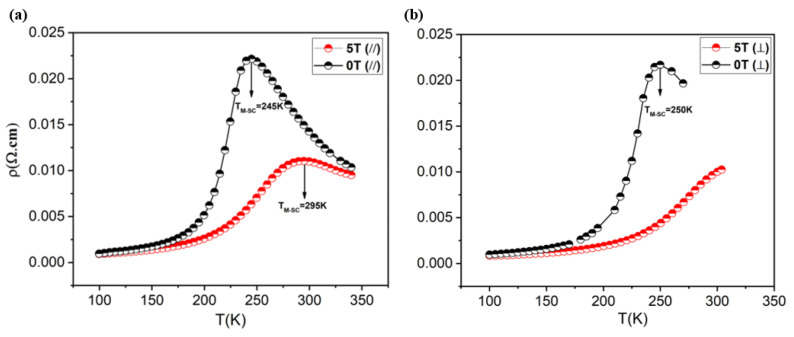
Temperature dependence resistivity of LBMTO-5 thin film in zero field and in a magnetic field of 5 T induction, applied parallel (**a**) and perpendicular (**b**) to the *ab* plane, respectively.

Lightly doped manganese oxides with perovskite structures belong to a strong magnetic–electronic coupling system. In order to explore the assessment of the relationship between the structural and physical properties of the films, the magneto-transport properties were exploited. In Figure 4 are shown the resistivity curves for the LBMTO-5 /STO film in 0 T and 5 T induction field applied in parallel (H// *ab* plane of the film) and perpendicular (H⊥ *ab* plane of the film) directions, respectively. The current always flows in the plane of the film, along the magnetically easy axis that corresponds to the [010] crystallographic direction. For both configurations, the resistivity (ρ-T) displays a ferromagnetic metallic (M) behavior at low temperatures which is transformed into a paramagnetic semiconductor (SC) (*i.e.,* dρ/dT < 0) at high temperatures. The next paragraphs will analyze in detail these two different behaviors.

A low residual resistivity of 9.64 × 10^−4^ Ω.cm is obtained at 100 K in zero-field that confirms the good quality of the film, in agreement with HRXRD results (see Section 3.1.). The maxima of the curves in Figure 4 clearly define the transition metal–semiconductor transition temperature T_M-SC_, which has a value close to the T_C_. This suggests a strong interplay between the magnetic and transport properties in the LBMTO-5 film. It should be noted that the maximum resistivity, and thus the T_M-SC_, shifts to a higher temperature with the increasing magnetic field. This behavior is in accordance with a delocalization of polarons that is characteristic of double-exchange ferromagnets. Moreover, a crystallographic strain present in the film is expected to reduce the resistivity and to shift the transition temperature T_M-SC_ toward higher temperatures with respect to their bulk counterpart [12].

In order to understand the charge transport mechanisms responsible for the conduction along the LBMTO-5 /STO structure, the Zener double exchange (ZDE) polynomial law [18,28] is employed in the low-temperature ferromagnetic metallic state corresponding to the temperature range of 98–175 K. The ZDE polynomial low has the form:

(1) $$\rho \left(T\right)=\rho_{0}+\rho_{2}T^{2}+\rho_{4.5}T^{4.5}$$

Here, ρ_0_ is the resistivity due to point-defect scattering; ρ_2_ represents the electrical resistivity due to the electron–electron scattering; ρ_4.5_ is the resistivity contributions due to electron–electron, electron–magnon and electron–phonon scattering processes; T^2^ and T^4.5^ are the corresponding temperature values. 

**Figure 5:**
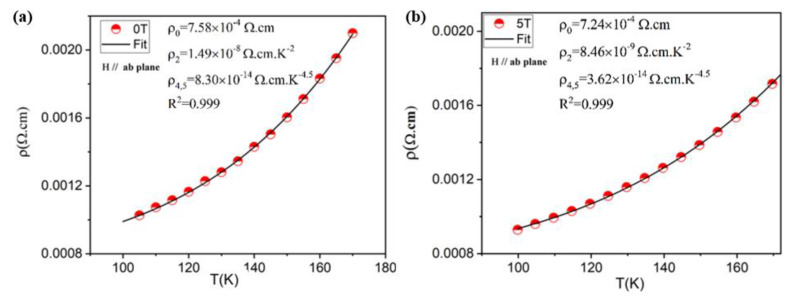
Fitting plots of the resistivity data of LBMTO-5 thin film in the low temperature ferromagnetic metallic state by using Equation (1) for a zero-field (**a**) and under 5 T induction (**b**), in the parallel geometry.

**Figure 6:**
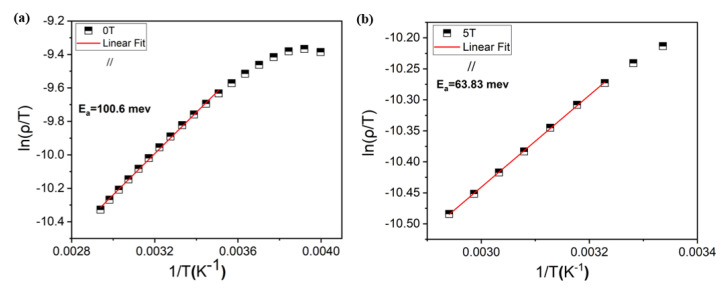
Fitting plots of the resistivity data of ln(ρ/T) as a function of 1/T of LBMTO-5 thin film in the paramagnetic semiconductor region by using Equation (2) for a zero-field (**a**) and under 5 T (**b**), in the parallel geometry.

The experimental data of Figure 4 are fitted according to the ZDE Equation (1) and the results for the parallel configuration are displayed in Figure 5 (for the perpendicular configuration see Supplementary Materials, Figure S4). The consistency of the fits is evident from the values of 0.999 of the correlation coefficient (R^2^). Moreover, the expected increase in the ρ_0_, ρ_2_ and ρ_4.5_ parameters in the bulk form [18] is mainly due to the scattering of charge carriers by the grain boundaries. In the paramagnetic semiconductor region, the (ρ-T)_//_ curve is usually described by the small polarons model [29,30]. This model has the form:

(2) $$\rho \left(T\right)=\mathrm{BT}\;\exp\frac{E_{a}}{k_{B}T}$$

where E_a_ is the activation energy for hopping conduction and B is the residual resistivity. The activation energy E_a_ and the model fitting is displayed in Figure 6. The obtained value of the E_a_ in the absence of an external magnetic field is lower than E_a_ deduced elsewhere in the bulk counterpart. A lower value of the E_a_ suggests that the polaron hopping becomes easier in the epitaxial thin film than in the bulk form.

**Figure 7:**
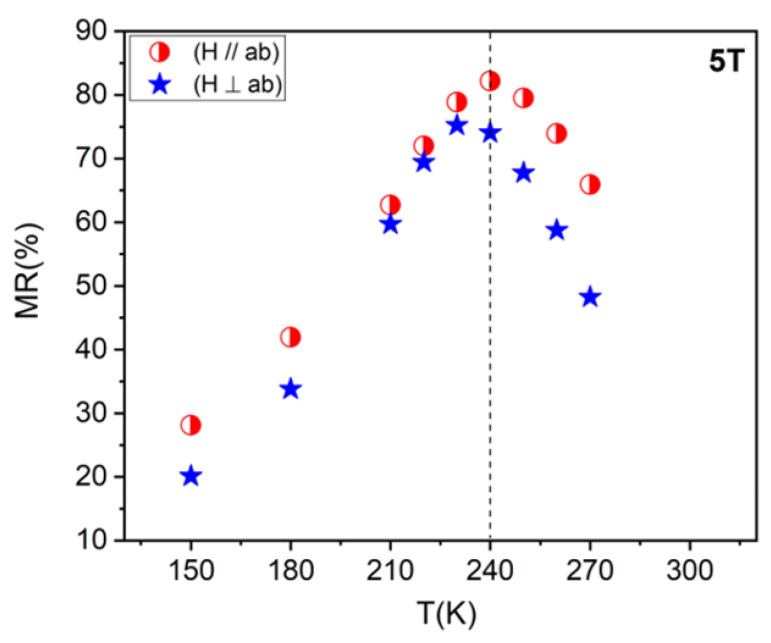
Temperature dependence of magnetoresistance MR of the LBMTO-5 thin film, measured under a magnetic field of 5 T along in-plane and out-of-plane geometry.

The magnetoresistance of the LBMTO-5/STO structure is determined according to the formula: $\mathrm{MR}=\left|\frac{R\left(H=0\right)-R\left(H\right)}{R\left(H\right)}\right|$, as shown in Figure 7. It can be observed that the higher values of MR are obtained when the magnetic field is applied in-plane parallel to the film (parallel geometry). As shown in Figure 7, the MR_//_ (for H = 5 T) reaches a maximum value of 82% at a temperature close to the T_C_. The LBMTO-5/STO epitaxial thin film has a larger MR in comparison with LBMTO-2/STO (MR (5 T) = 60% at 300 K), LCMO-STO (MR (6.8 T) = 32% at 272 K) and LCMO-LAO (MR (6.8 T) = 22.5% at 274 K) thin films, as reported by Egilmez et al. [12]. 

**Figure 8:**
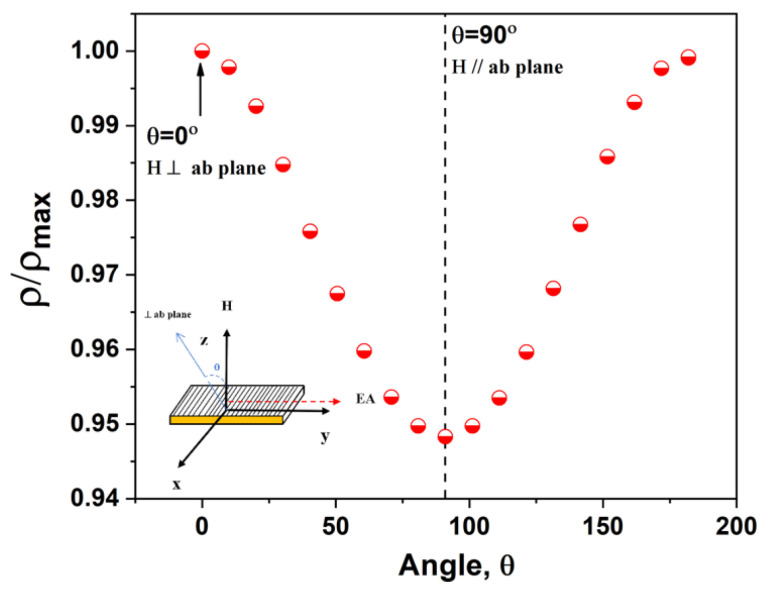
Angular dependence of the (ρ/ρ_max_) of LBMTO-5/STO system in a magnetic field of 5 T and at a temperature near the metal–semiconductor transition (250 K). Inset: schematic view of the measurement with the sample rotating in the in-plane (parallel) and the out-of-plane (perpendicular) geometry.

To gain more insight into the physical mechanism of the magnetoresistance anisotropy observed for our LBMTO-5/STO epitaxial films, the normalized resistivity (ρ/ρ_max_) is measured as a function of the angle θ between the applied field H and the *ab* plane. Note that here, the θ = 0° corresponds to the configuration where the sample is perpendicular to the direction of H, being equivalent to the out-of-plane or perpendicular geometry. Figure 8 shows a typical angular dependence of (ρ/ρ_max_) for LBMTO-5/STO structure at a temperature near the metal–semiconductor transition (250 K) and in a field of 5 T induction. 

In agreement with the initial expectations that were also confirmed by magnetic measurements (see Figure 2, Section 3.2.), a low resistivity value is obtained in the parallel configuration at θ = 90°, equivalent to the in-plane geometry that matches with a maximum of MR (see Figure 7), as compared to perpendicular configuration. Obviously, there is an in-plane anisotropy with the (ρ/ρ_max_)_⊥_-(ρ/ρ_max_)_//_ ≅ 9%. This can be easily understood because for the existing in-plane anisotropy, a more complete saturation of the magnetization can be achieved in the in-plane geometry (parallel configuration).

## 4. Conclusions

To summarize, an epitaxial LBMTO-5 epilayer was successfully grown on a STO(001) substrate. The magnetic, magnetocaloric effect and magnetoresistance of the LBMTO-5 film with a thickness of 97 nm have been studied at different directions of the applied magnetic field with respect to the sample plane. Herein, the epilayer exhibits a second-order FM–PM phase transition around 234 K and an in-plane magnetic uniaxial easy axis (along the direction of the longer edge of the sample). Further, at increasing temperature, a gradual metal to semiconductor transition finishing at 245 K is observed. The carriers transport belongs to a small polaron hopping model in paramagnetic semiconductors. Under 5 T magnetic field applied parallel to the film surface, the maximum of the (−ΔS_M_) and of the relative cooling power RCP are 17.27 mJ/cm^3^ K and 1400 mJ/cm^3^ K, respectively. These results highlight the potential applications of the present films for micro-scale magnetic cooling. Another important finding is that the LBMTO-5/STO epitaxial thin film has a giant magnetoresistance as high as 82% at a temperature close to the T_C_, which may be interesting for electro-magnetic applications. A magnetoresistance anisotropy reaching a maximum value around T_C_ is also characteristic of such films.

## Data Availability

Not applicable.

## Supplementary Materials

The following supporting information can be downloaded at: https://www.mdpi.com/article/10.3390/ma15228003/s1, Figure S1: Schematic diagrams of the in-plane lattice arrangement for LBMTO-5/STO(001); Figure S2: The Arrott plot of μ_0_H/M versus M^2^; Figure S3: Magnetic entropy change versus the (μ_0_H/T_C_)^2/3^; Figure S4: Fitting plots of the resistivity data in the low temperature ferromagnetic metallic state in the perpendicular geometry.
