# Supplementary material for: Magnetocaloric and Giant Magnetoresistance Effects in La-Ba-Mn-Ti-O Epitaxial Thin Films: Influence of Phase Transition and Magnetic Anisotropy
Source: Materials (Basel). 2022 Nov 12;15(22):8003. doi: 10.3390/ma15228003 (PMC9699288; doi:10.3390/ma15228003)
Supplement: Supplementary file 1 [file materials-15-08003-s001.zip › materials-1993705-supplementary.pdf]

Supplementary Information

Magnetocaloric and giant magnetoresistance effects in La–Ba–Mn-Ti-O epitaxial thin films: Influence of phase transition and magnetic anisotropy.

Marwène Oumezzine[1,*], Cristina Chirila[2], Iuliana Pasuk[2], Aurelian Catalin Galca[2], Aurel Leca[2], Bogdana Borca[2,*], Victor Kuncser [2,*]

*Correspondence: oumezzine@hotmail.co.uk (Marwène Oumezzine), bogdana.borca@infim.ro (Bogdana Borca), kuncser@infim.ro (Victor Kuncser),

[1]Laboratoire de Physico-Chimie des Matériaux, Université de Monastir, 5000 Monastir, Tunisia;
[2]National Institute of Materials Physics, 077125 Magurele, Romania;

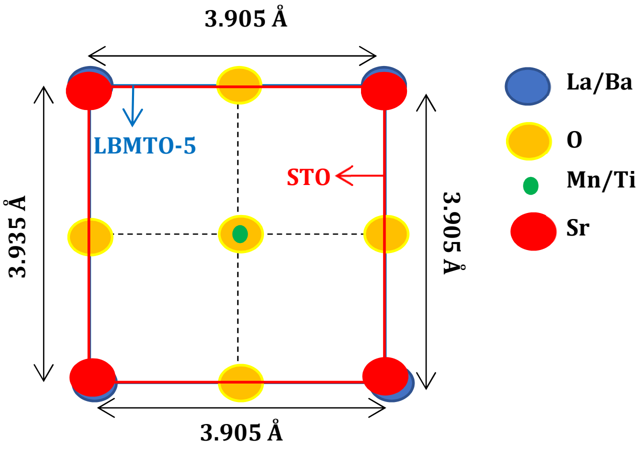

Figure S1: Schematic diagrams of in-plane lattice arrangement for LBMTO-5 on STO (001).

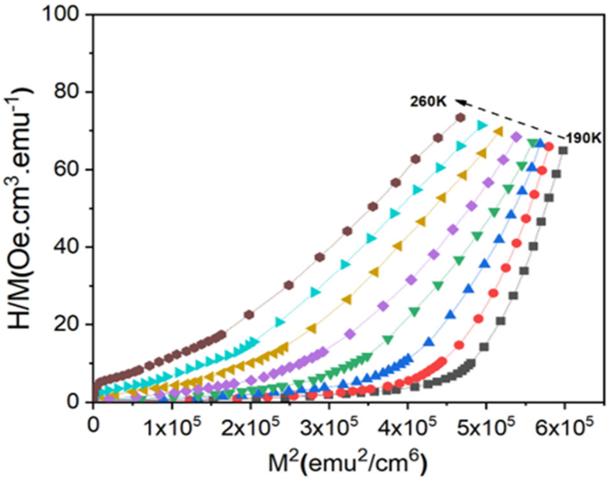

Figure S2: The Arrott plot of μ_0H/M versus M^2 represented from the isothermal magnetization curves measured at different temperatures around T_C for LBMTO-5 on STO (001).

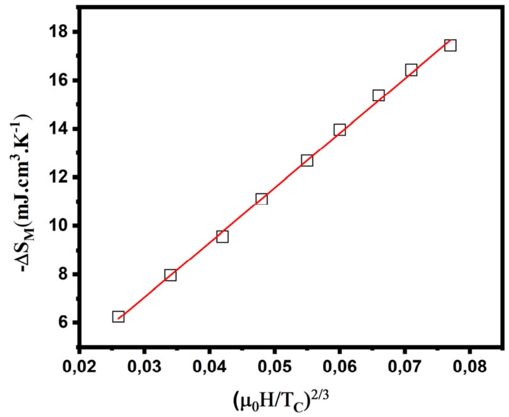

Figure S3: Corresponding plot and the linear fitting (red line) of the magnetic entropy change on the parameter $(\mu_0 H/T_C)^{2/3}$ of of LBMTO-5 thin film in agreement with the existence of long-range ferromagnetic interactions in epitaxial LBMTO-5 thin films.

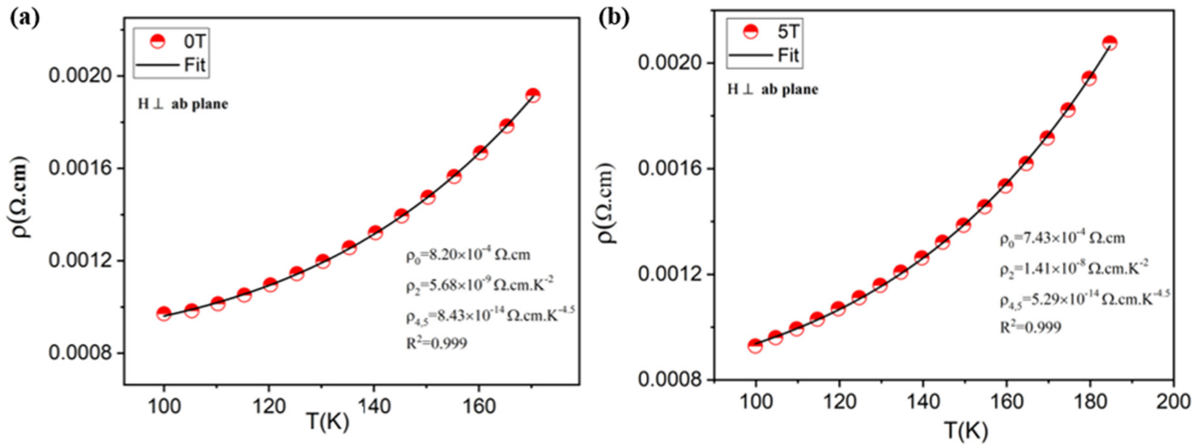

Figure S4: Fitting plots of the resistivity data of LBMTO-5 thin film in the low temperature ferromagnetic metallic state by using equation (1) $\rho(T)=\rho_0+\rho_2 T^2+\rho_{4.5} T^{4.5}$ for a zero-field (a) and under 5 T (b) in the perpendicular geometry.
